# Supplementary material for: A Shadowing Problem in the Detection of Overlapping Communities: Lifting the Resolution Limit through a Cascading Procedure
Source: PLoS One. 2015 Oct 13;10(10):e0140133. doi: 10.1371/journal.pone.0140133 (PMC4603673; doi:10.1371/journal.pone.0140133)
Supplement: S1 Table — (PDF) [file pone.0140133.s001.pdf]

Table S1: Description and properties of real networks used in this study.

Network	N^a	L^b	L'^c	L''^d	$\langle k \rangle^e$	C^f	Reference
arXiv	30 561	125 959	124 930	121 006	8.24	0.63	[Palla 2005]
Email	1 134	5 143	5 143	5 055	9.07	0.46	[Guimera 2003]
Gnutella	36 682	88 328	88 303	4 056	4.82	0.01	[Ripeanu 2002]
Internet	22 963	48 436	48 436	24 170	4.22	0.23	[Hébert-Dufresne 2011]
PGP	10 680	24 316	24 316	17 135	4.55	0.27	[Boguñá 2004]
Power	4 941	6 594	6 594	1 371	2.67	0.08	[Watts 1998]
Protein	2 640	6 379	6 296	3 721	4.83	0.21	[Palla 2005]
Words	7 207	31 784	31 784	22 169	8.82	0.15	[Palla 2005]

[a] Number of nodes.

[b] Number of links. We use an undirected projection where all multi-edges are only counted once, and where self-loops are removed.

[c] Number of *assignable* links for the GCE algorithm, i.e. links that are part of a component which contains at least one 3-clique.

[d] Number of *assignable* links for the CPA, i.e. links that are part of at least one 4-clique.

[e] Average degree, with $\langle k \rangle = 2L/N$.

[f] Average clustering coefficient, i.e. average fraction of existing edges between neighbors of a node.
