# Supplementary material for: A Shadowing Problem in the Detection of Overlapping Communities: Lifting the Resolution Limit through a Cascading Procedure
Source: PLoS One. 2015 Oct 13;10(10):e0140133. doi: 10.1371/journal.pone.0140133 (PMC4603673; doi:10.1371/journal.pone.0140133)
Supplement: S2 Table — (PDF) [file pone.0140133.s002.pdf]

Table S2: Summary of the results presented in Fig. 5. and Fig. 6.

	arXiv	Email	Gnutella	Internet	PGP	Power	Protein	Words
CPA_s [a]	54.3	74.8	0	95.9	47.0	79.2	45.9	50.6
CPA_f [b]	7.7	65.8	0	88.0	6.7	5.5	18.1	22.0
Iterations [c]	3	2	1	4	2	2	2	2
ΔT [d]	1.76	2.12	1.00	1.11	1.00	3.60	1.58	1.70
GCE_s [a]	26.9	64.9	99.7	40.1	32.4	92.4	51.7	54.5
GCE_f [b]	2.9	12.1	84.9	9.6	12.5	63.0	21.6	14.9
Iterations [c]	8	6	4	6	5	4	6	8
ΔT [d]	1.76	1.08	8.69	1.11	1.56	8.08	2.69	3.02
LCA_s [a]	22.3	47.7	35.3	28.2	46.3	63.6	38.5	46.1
LCA_f [b]	2.3	1.3	18.7	2.7	4.5	4.4	6.2	2.6
Iterations [c]	7	5	2	5	6	3	3	5
ΔT [d]	1.20	1.16	1.38	1.05	1.41	2.05	1.39	1.51

[a] Percentage of remaining assignable links for a *standard* use of the algorithm.
[b] Percentage of remaining assignable links after the cascading approach is applied.
[c] Number of applications of the cascading algorithm before the final state is reached.
[d] Relative increase in running time of the complete algorithm, averaged over 10 independent realizations, timed at the millisecond precision.
