# Supplementary material for: A Shadowing Problem in the Detection of Overlapping Communities: Lifting the Resolution Limit through a Cascading Procedure
Source: PLoS One. 2015 Oct 13;10(10):e0140133. doi: 10.1371/journal.pone.0140133 (PMC4603673; doi:10.1371/journal.pone.0140133)
Supplement: S3 Table — (PDF) [file pone.0140133.s003.pdf]

Table S3: Description and properties of real networks used in this study.

Network	N^a	L^b	$\langle k \rangle^c$	C^d	g^e	$\langle s \rangle^f$	$\langle \rho \rangle^g$	Reference
Amazon	334 863	925 872	5.53	0.40	271 570	11.7	0.77	[Yang 2015]
DBLP	317 080	1 049 866	6.62	0.63	13 477	53.4	0.52	[Yang 2015]
YouTube	1 134 890	2 987 624	5.27	0.08	16 386	7.88	0.73	[Yang 2015]

[a] Number of nodes.
[b] Number of links. An undirected projection without multi-edges and self-loops is used.
[c] Average degree, with $\langle k \rangle = 2L/N$.
[d] Average clustering coefficient, i.e. average fraction of existing edges between neighbors of a node.
[e] Number of structural communities
[f] Average size, in nodes, of functional communities
[g] Average density of functional communities
