# Supplementary material for: A Shadowing Problem in the Detection of Overlapping Communities: Lifting the Resolution Limit through a Cascading Procedure
Source: PLoS One. 2015 Oct 13;10(10):e0140133. doi: 10.1371/journal.pone.0140133 (PMC4603673; doi:10.1371/journal.pone.0140133)
Supplement: S4 Table — (PDF) [file pone.0140133.s004.pdf]

Table S4: Summary of the results presented in Fig. 9.

Network	LCA_s [a]	LCA_f [b]	It. [c]	ΔT [d]	ΔNMI [e]
Amazon	27.1	2.9	6	1.28	0.156
DBLP	20.3	2.8	8	1.19	-0.016
YouTube	66.0	2.6	10	1.47	1.128

[a] Percentage of remaining assignable links for a *standard* use of the algorithm.
[b] Percentage of remaining assignable links after the cascading approach is applied.
[c] Number of applications of the cascading algorithm before the final state is reached.
[d] Relative increase in running time of the complete algorithm, averaged over 10 independent realizations, timed at the millisecond precision.
[e] Relative change in normalized mutual information.
