# Supplementary material for: A Shadowing Problem in the Detection of Overlapping Communities: Lifting the Resolution Limit through a Cascading Procedure
Source: PLoS One. 2015 Oct 13;10(10):e0140133. doi: 10.1371/journal.pone.0140133 (PMC4603673; doi:10.1371/journal.pone.0140133)
Supplement: S5 Table — (PDF) [file pone.0140133.s005.pdf]

Table S5: Summary of the results presented in Fig. 10.

μ^a	$\langle\{\Delta\text{NMI}\}\rangle^b$	$\sigma(\{\Delta\text{NMI}\})^c$	$\min(\{\Delta\text{NMI}\})^d$	$\max(\{\Delta\text{NMI}\})^e$
0.1	0.023	0.29	-0.17	4.79
0.6	0.018	0.31	-0.11	2.25

[a] Mixing parameter of the LF network.
[b] Average relative difference in NMI.
[c] Standard deviation of the distribution of relative difference in NMI.
[d] Minimum of the distribution of relative difference in NMI.
[e] Maximum of the distribution of relative difference in NMI.
