# Supplementary material for: A Shadowing Problem in the Detection of Overlapping Communities: Lifting the Resolution Limit through a Cascading Procedure
Source: PLoS One. 2015 Oct 13;10(10):e0140133. doi: 10.1371/journal.pone.0140133 (PMC4603673; doi:10.1371/journal.pone.0140133)
Supplement: S6 Table — The mixing parameter is given by the average of the ratio 1-ki(in)/ki taken over all nodes i, where ki(in) is the number of neighbors of node i which share at least a community with i, and where k i is the degree of node i[30]. The coefficient of variation c v is defined as the standard deviation of a distribution, normalized by the mean. (PDF) [file pone.0140133.s006.pdf]

Table S6: Properties of the detected community structure of real networks.

		arXiv	Email	Gnutella	Internet	PGP	Power	Protein	Words
CPA	$\hat{\mu}^a$	0.05	0.12	0.06	0.00	0.02	0.01	0.07	0.22
	$c_v{}^b$	0.56	1.11	0.43	0.73	1.16	0.33	0.83	1.27
	$\langle\Omega\rangle^c$	1.69	1.47	1.13	1.24	1.15	1.15	1.15	1.49
GCE	$\hat{\mu}^a$	0.11	0.11	0.00	0.07	0.03	0.01	0.09	0.24
	$c_v{}^b$	1.03	0.15	0.44	1.85	1.25	0.51	0.94	0.72
	$\langle\Omega\rangle^c$	1.54	1.82	1.02	1.22	1.20	1.12	1.09	1.59
LCA	$\hat{\mu}^a$	0.08	0.23	0.12	0.05	0.08	0.08	0.12	0.25
	$c_v{}^b$	0.74	2.42	0.89	5.51	0.78	0.48	0.70	0.91
	$\langle\Omega\rangle^c$	2.96	2.85	1.69	1.94	2.13	1.28	1.80	2.84

[a] Estimated mixing parameter.

[b] Coefficient of variation of the size distribution.

[c] Average membership number.
