# Supplementary figures and images for: A Shadowing Problem in the Detection of Overlapping Communities: Lifting the Resolution Limit through a Cascading Procedure
Source: PLoS One. 2015 Oct 13;10(10):e0140133. doi: 10.1371/journal.pone.0140133 (PMC4603673; doi:10.1371/journal.pone.0140133)

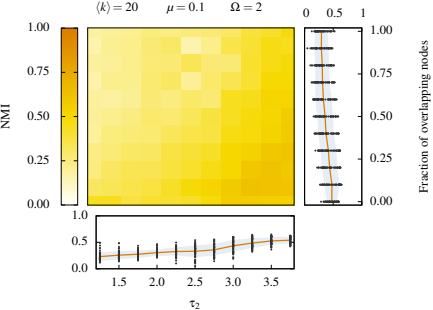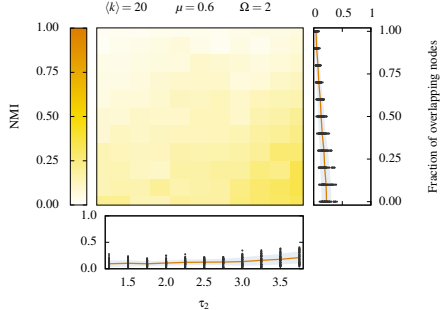

Supplement: S1 Fig — This figure shows the average value of the NMI before any further detection steps are performed, i.e. it illustrates the results of the pure LCA. (Left panel) Lightly mixed LF networks with a mixing parameter μ = 0.1. (Right panel) Heavily mixed LF networks μ = 0.6. We use networks of N = 5 000 nodes of average degree 〈k〉 = 20, that belongs to Ω = 2 communities (if they overlap). (PDF) [file pone.0140133.s007.pdf]

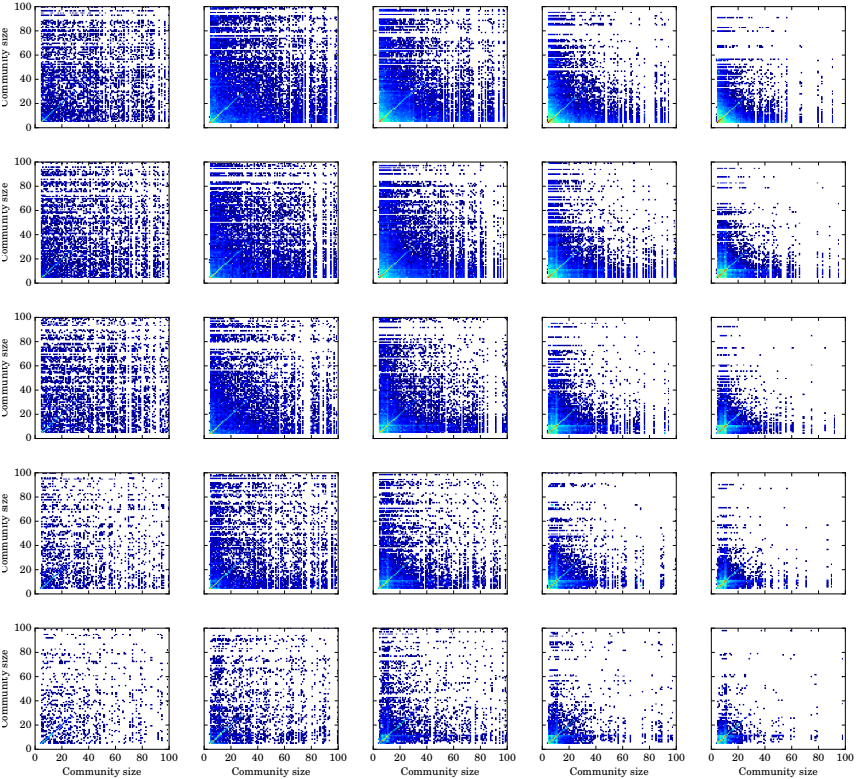

Supplement: S2 Fig — This array samples 25 points of the parameter space, i.e. fractions of overlapping nodes set to f = [1/5, 2/5, 3/5, 4/5, 1] (bottom to top) and size distribution exponents set to τ 2 = [1.5, 2, 2.5, 3, 3.5] (left to right). All networks consists of N = 5 000 nodes of average degree 〈k〉 = 20, that belongs to Ω = 2 communities (if they overlap), with a mixing parameter μ = 0.1. Each subplot shows the number of communities of x nodes in the neighborhood of communities of y nodes, for all x, y ≤ 100. Communities that share at least one node are defined as neighbors. In all cases, large communities are found in the vicinity of smaller communities of all sizes (off-diagonal elements are present). This pattern is however more pronounced in the highly heterogeneous region (right-hand side of the figure), where shadowing occurs (See Fig 10 of main text). The correlation patterns are essentially the same in the high mixing case μ = 0.6 (not shown). (PDF) [file pone.0140133.s008.pdf]

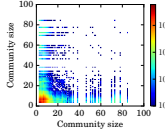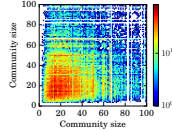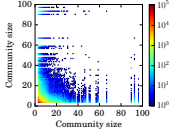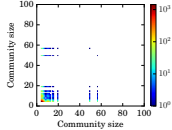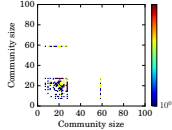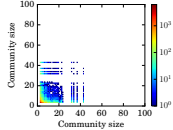

(a) arXiv

(b) Email

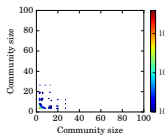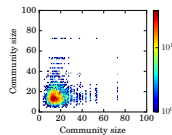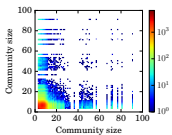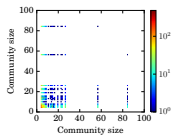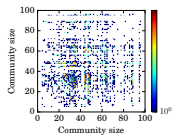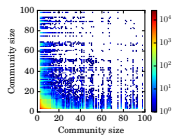

(c) Gnutella

(d) Internet

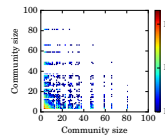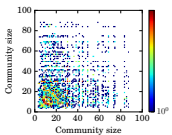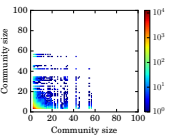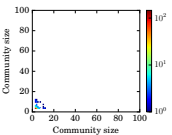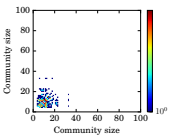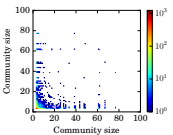

(e) PGP

(f) Power

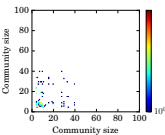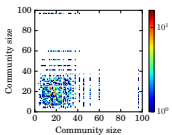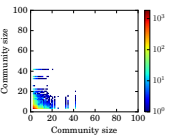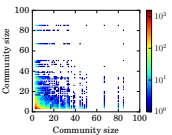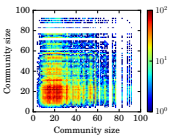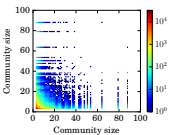

(g) Protein

(h) Words

Supplement: S3 Fig — For each network, correlations are computed using the community structure detected by the cascading version of CPA (left), GCE (center) and LCA (right). Each subplot shows the number of communities of x nodes in the neighborhood of communities of y nodes, for all x, y ≤ 100. Communities that share at least one node are defined as neighbors. To identify the possibility of shadowed communities, one must look for large communities in the vicinity of small ones, i.e. for off diagonal elements in the rows / columns corresponding to community sizes that are present in the network. For CPA, one need not look far away from the diagonal, since small communities can shadow even smaller communities (see Fig 1 of the main text). For the other two algorithms, larger differences in sizes are required (see Figs 2 and 4 of the main text). These observations suggests that shadowing occurs in all cases (well populated correlation diagrams). (PDF) [file pone.0140133.s009.pdf]
